# Non-specific symptoms as clues to changes in emotional well-being

**DOI:** 10.1186/1471-2296-12-77

**Published:** 2011-07-26

**Authors:** Andre Matalon, Andy Kotliroff, Gari Blumberg, John Yaphe, Eliezer Kitai

**Affiliations:** 1 Department of Family Medicine, Faculty of Medicine, Tel-Aviv University, Ramat Aviv, 69978, Israel; 2 Department of Family Medicine, Clalit Health Services, Rabin Medical Center, Beilinson Hospital, Petach Tikva 49100 Israel; 3 Department of Family Medicine, Leumit Health Services, 23 Shprintzak Street, Tel Aviv, Israel; 4 Community Health, School of Health Sciences, University of Minho, Braga, Portugal

**Keywords:** somatic, non-specific symptoms, depression, anxiety

## Abstract

**Background -:**

Somatic symptoms are a common reason for visits to the family physician. The aim of this study was to examine the relation between non-specific symptoms and changes in emotional well-being and the degree to which the physician considers the possibility of mental distress when faced with such patients.

**Methods -:**

Patients who complained of two or more symptoms including headache, dizziness, fatigue or weakness, palpitations and sleep disorders over one year were identified from the medical records of a random sample of 45 primary care physicians. A control group matched for gender and age was selected from the same population. Emotional well-being was assessed using the MOS-SF 36 in both groups.

**Results -:**

The study group and the control group each contained 110 patients. Completed MOS questionnaires were obtained from 92 patients, 48 patients with somatic symptoms and 44 controls. Sixty percent of the patients with somatic symptoms experienced decreased emotional well being compared to 25% in the control group (*p *= 0.00005). Symptoms of dizziness, fatigue and sleep disturbances were significantly linked with mental health impairments. Primary care physicians identified only 6 of 29 patients (21%) whose responses revealed functional limitations due to emotional problems as suffering from an emotional disorder and only 6 of 23 patients (26%) with a lack of emotional well being were diagnosed with an emotional disorder.

**Conclusions -:**

Non-specific somatic symptoms may be clues to changes in emotional well-being. Improved recognition and recording of mental distress among patients who complain of these symptoms may enable better follow up and treatment.

## Background

Somatic symptoms are the main reason for visits to the family physician in primary-care clinics [1-5]. Often the visit yields no clear etiology or diagnosis that can explain the symptoms and the physician may describe it as "idiopathic," "atypical," "functional," or "non-specific" [3]. A broad study regarding symptoms in primary care [4] revealed that only 16% of the symptoms presented had a clear, organic etiology; for 74% no etiology was established over a three-year follow-up period, and the symptom remained the diagnosis. Frequently symptoms will pass without any treatment, and very often people do not even consider visiting their physician when symptoms first appear.

Although not tied to a definite diagnosis, non-specific symptoms can interfere with patients' daily routine and be as detrimental to their quality of life as can organic and mental diseases that have been diagnosed unequivocally [5-8]. For example, decreased functioning from chronic fatigue is identical to that observed in patients with unbalanced hyperthyroidism or in those recovering from myocardial infarction [9].

Lasting non-specific somatic symptoms initiate a "journey of clarification." The co-travelers on this costly journey are the patient, who wants to know "what's wrong with me," and the physician, who wants to rule out treatable organic diseases. The journey includes repeated visits to primary care physicians, countless lab tests, many medications and other treatments, as well as referrals to a variety of specialists [10-12]. In addition, it is well known that mental stress, difficult life events and somatization, of course, can be experienced as somatic symptoms. At times, the symptoms can be the only expression of emotional distress or a psychiatric disease [13-15].

The two psychiatric diseases that family physicians most frequently encounter in the primary care clinic are depression and anxiety [16]. A Dutch study [17] looked at medically unexplained symptoms in the elderly and found a high rate of co-morbidity with anxiety and depression. Often these disorders are not diagnosed or treated [16,18-22] and cause reduced daily functioning and an over-use of health services. Frequently they present as somatic symptoms, and disregarding them may deepen their hold and harm doctor-patient relations [16].

The aim of this study was to examine the relation between non-specific symptoms and changes in emotional well-being such as depression and anxiety among patients consulting their primary care physician. The study also examined whether the clues of mental distress become stronger with an increase in the number of the non-specific symptoms and also the degree to which primary physicians consider mental distress when faced with a patient who complains of these symptoms. Our hypothesis was that multiple non-specific somatic symptoms are predictive of emotional distress, specifically anxiety and depression, and that this tends to be under-diagnosed by general practitioners.

## Methods

This was a cross sectional survey, conducted in the community among individuals insured by one of Israel's four HMOs (health maintenance organizations). Patients were identified from the medical records and clues for anxiety and depression were assessed by a questionnaire at one point in time.

Data were derived from patients' medical files and from questionnaires. We obtained approval form the Research Regulation Committee of the HMO (Leumit Health Services) to use the data. Ethical approval for our study was granted by the Ethics Committee of Tel Aviv University.

### Research population

This study, performed in 2002, examined the last 60 patient-doctor meetings (consultations) of a random sample of 45 physicians selected from among all the primary-care physicians in the HMO. Included were 15 general practitioners, 15 specialists in family medicine, and 15 internists. Data regarding the consultations were gathered using the computer program which the HMO uses for maintaining medical records.

A total of 2,700 medical consultations were examined. The patients were men and women, 20-70 years old. Patients with active cancer, dementia, congestive heart failure and those under psychiatric care or on dialysis were excluded from the sample. The symptoms noted were headache, dizziness, fatigue or weakness, palpitations and sleep problems. The research group consisted of 110 patients who complained of two or more of these symptoms at one visit or who complained of one symptom in more than two consultations within the year prior to the last meeting.

The control group consisted of individuals who were matched to each person in the research group for gender and age (+/-5 years) and who had visited the same doctor on the same day, but did not have the symptoms of which the member of the research group complained.

### Research procedure

The MOS-SF 36 Questionnaire, which measures patients' perception of their physical and mental health and social functioning, was used to assess the mental state of participants in both groups [23-25]. Two domains in the questionnaire which relate to emotional well-being and limitation of function due to emotional distress and which reflect the study hypothesis were selected as the outcome measures of this study. A previously validated Hebrew version of the questionnaire was mailed to the participants' homes [26] with an accompanying letter and stamped, self-addressed envelope for sending the replies to the HMO headquarters. A month after the questionnaires had been sent, those who had not yet returned them received a telephone reminder and a new questionnaire was mailed to them on request. One month later, another round of telephone calls was made to those who had not yet returned the questionnaires.

### Data processing

Results were processed in accordance with the procedures detailed by Hays and Sherbourne in the MOS-SF 36 questionnaire [25] according to which a low score suggests that the patient suffers from a mental health problem. Patients whose score is lower than 50 on a 0-100 scale on questions of emotional well being and functional limitations due to emotional problems are at high risk for problems in these areas.

Statistics were calculated using BMDP Statistical Software (1993) and Epi-Info.

Correlation between each of the five symptoms and functional limitations due to emotional problems and emotional well being was assessed using the chi-squared statistic.

Correlation between the number of somatic symptoms of which the patient complained and functional limitation due to emotional problems and emotional well being was calculated using the chi squared statistic.

The chi squared test was also used to compare ratios between the research and control groups.

After retrieving from the questionnaires those patients who were at high risk to suffer from emotional problems or from lack of emotional well being, the researchers then checked to see whether the physician listed a mental health disorder as a possible diagnosis. The medical records of the patients in the control group were not examined to see if their physicians had later recorded mental health disorders.

Statistical significance was set at the level of p < 0.05.

## Results

The researchers surveyed 2,700 consultations from the computerized medical records of 45 primary physicians. Of the total research consultations, 1,545 meetings were with women and 1,155 with men. The examination of these consultations yielded 110 patients who complained of functional somatic symptoms (Research Group), and 110 patients were matched to form the Control Group (see Methods).

The symptom that was most frequent among the 110 patients in the research group from the review of the electronic medical records was headache (N = 32, 29.1%), followed by fatigue (N = 29, 26.3%) and dizziness (N = 27, 24.5%). Twenty patients in this group of 110 (18.2%) had a single symptom. Complaints of two of the five symptoms were voiced by 46 patients (41.8%), 32 patients (29%) complained of three symptoms and twelve (10.9%) of four while no patients complained of all five.

After telephone calls and repeated reminders, 92 completed MOS-SF 36 questionnaires were returned (41.8%), of which 48 were from the research group (43.6% of questionnaires sent) and 44 from the control group (40% of questionnaires sent).

Of the remaining 128 questionnaires, 8 were returned by the postal service (addressee unknown), 17 arrived without demographic details, rendering them unusable, and 103 questionnaires were not returned at all. Table 1 presents the demographic characteristics of the study sample, showing no significant differences between responders in the cases and control group by age, gender, marital status or number of children.

**Table 1:** Demographic characteristics of the study population in the study of somatic symptoms and emotional well being (n = 92)

	Research Group (N = 110)		Control Group (N = 110)		
**All respondents (%)**	**Total = 110**	**Responders = 48 (43.6%)**	**Total = 110**	**Responders = 44 (40%)**	**Comparison of research and control groups**

Number of women (%)	79 (71.8)	34 (70.3)	79 (71.8)	34 (77.3)	P = 0.3, NS

Number of men (%)	31 (28.2)	14 (29.7)	31 (28.2)	10 (22.7)	P = 0.3, NS

Mean age in years (range, s.d.)	46.5 (21-69)	51.1 (23-69, 11.7)	46.6 (20-70)	53.1 (24-70, 11.4)	P = 0.4, NS

Marital status (% married)		63%		62%	P = 0.8, NS

Mean number of children		2.1		2.3	P = 0.7, NS

Two domains from the MOS SF-36 questionnaire were addressed - functional limitations due to emotional problems and emotional well being. A score of 0 to 100 is possible. A lower score indicates more distress.

Table 2 presents the results of the questionnaire scores. Sixty percent of the patients in the research group suffered from lack of emotional well being (with a score less than 50), while in the control group the rate was 25.0% (*p *= 0.00005). Forty five percent of the patients in the research group suffered from functional limitations due to emotional problems as compared to 43% of the patients in the control group (*p *= 0.48, n.s.).

**Table 2:** MOS SF-36 scores for emotional well being and for limitation of function due to emotional distress for the research and control groups (n = 92)

	Research group n = 48	Control group n = 44	
Mean MOS score for emotional well-being (median, s.d.)	42.3 (33, 44.9)	76.1 (100, 39.5)	P = 0.0003

%of patients with impaired emotional well-being (MOS score < 50)	60%	25%	P < 0.0005

Mean MOS score for functional limitation due to emotional factors (median, s, d)	54.1 (56, 22.0)	55.6 (58, 22.0)	P = 0.7, NS

% of patients with impaired functional limitation due to emotional factors (MOS score < 50)	45%	43%	P = 0.48, NS

The mean score for emotional well being was lower in the research group (a score of 42.3, as compared to the control group score of 76.1; *p = *0.0003). There was no significant difference found in functional limitations due to emotional problems (mean score for cases 54.1; mean score in control group 55.6, *p = *0.7, n.s.).

Comparisons between the research and control groups with regard to functional limitations (MOS Score < 50 for functional limitation) by individual symptoms are given in Table 3. There were no significant differences found regarding functional limitation when the two groups were compared by the presence of individual symptoms (headache, insomnia, dizziness, palpitations or fatigue).

**Table 3:** Comparisons between research and control groups with regard to presence of symptoms and functional limitations (MOS Score < 50 for functional limitation)

complaint	N with complaint	% with functional limitations		Statistical significance
		**Research group**	**Control group**	

headache	32	46.9	43.3	P = 0.45, NS

fatigue	29	48.3	42.9	P = 0.39 NS

dizziness	28	53	40	P = 0.17, NS

palpitations	8	50	44	P = 0.51, NS

insomnia	15	53.3	42.9	P = 0.32, NS

Comparisons between the research and control groups with regard to a lack of emotional well-being (MOS Score < 50 for emotional well-being) by individual symptoms are given in Table 4. There were significant differences found between the proportion of patients with a lack of emotional well-being in the research group for four of the five symptoms studied (headache, insomnia, dizziness, and fatigue). There was no significant difference in emotional well-being for patients with palpitations compared to the control group.

**Table 4:** Comparisons between research and control groups with regard to presence of symptoms and lack of emotional well-being (MOS Score < 50 for emotional well-being)

complaint	N with complaint	% with lack of emotional well-being		Statistical significance
		**Research group**	**Control group**	

headache	32	62.5	33.3	p = 0.0067

fatigue	29	65.5	33.3	P = 0.0038

dizziness	28	60.7	35.9	P = 0.024

palpitations	8	37.5	44	P = 0.51, NS

insomnia	15	66.7	39.0	p = 0.045

Among patients who complained of one symptom, 16.7% suffer limitations due to emotional problems. Among patients who complained of two symptoms, this rate is 60.0%, which is significantly higher than the rate among those with one symptom (*p <*0.05). Among the patients who reported one symptom the percentage of patients with low emotional well being was the lowest (33%), and went up to 83.7% among patients with two symptoms (p < 0.01). No correlation could be made between the number of symptoms greater than two and increased functional limitations or lack of emotional well being.

Finally, among patients whose responses revealed functional limitations due to emotional problems (*N = *29 - out of 48 total), only six (20.7%) received a psychological diagnosis from their physicians: two were diagnosed with depression, two with anxiety, and two with stress. At the same time, among those patients whose responses revealed a lack of emotional well being (*N = *23), also six (26.1%) received a mental diagnosis: two were diagnosed with depression, three with anxiety, and one with stress. The medical records of the patients in the control group were not examined to determine the prevalence of recording of mental health diagnoses so a comparison of rates between the two groups could not be made.

## Discussion

Our research supports the hypothesis that patients who complain about nonspecific somatic symptoms suffer more from emotional distress, as seen by decreased emotional well being. It was surprising that such patients do not suffer significantly more from functional limitations due to emotional problems. Our study is a retrospective one which makes it difficult to ascertain whether the somatic symptoms cause the emotional distress or whether the opposite is true. As in most other studies on this subject, we can only say that there is a relationship but can not determine cause and effect.

As a tool for examining this relationship, we selected the MOS SF-36 Questionnaire, mostly for its "friendly" qualities in reference to a generally healthy population that sees a family physician for various reasons and its ability to assess a patient's functional state and health which has been proven reliable in previous studies [23-25].

The relationship between non-specific somatic symptoms, depression, and anxiety has been suggested in previous studies [2,16,27-29]. A perception of poor health, pain, or disability has been correlated with anxiety and depression symptoms in primary care patients [30]. 50%-80% of patients suffering from depression and anxiety first pay a visit to the family physician because of somatic complaints, without expressing any mental complaints [3,21,31,32]. Kroenke's work demonstrated that the rate of psychiatric disorders among patients who complain of somatic symptoms is at least twice as high as that in the general population of primary care clinic patients [6,16].

Studies have tried to characterize the patients who come to a physician for somatic symptoms such as headache, fatigue, and gastrointestinal complaints as compared to those who do not turn to a physician when these symptoms appear [33,34]. Findings indicate that those in the first group had suffered more stressful events throughout their lives, and were closer to meeting *DSM-IV *criteria for anxiety disorders or depression disorders. It seems that stressful events increase the negative perception of symptoms. Dirkswager and Verhaak [35] found that patients with medically unexplained symptoms were more likely to have psychological distress, functional impairment and social isolation.

Certain somatic symptoms may serve as indicators of emotional distress, and their appearance alerts the physician in a more pronounced way than do others. Headache, insomnia, fatigue and dizziness were found in our research to be potential indicators of emotional distress. Kroenke et al. also described these symptoms as being strongly linked to depression and anxiety [2]. Sloane's survey, too, shows that dizziness is a very common symptom in the primary care clinic and in a large number of cases is related to psychiatric disorders [36]. Haug, et al, did a large study in Norway and found associations between depression, anxiety and certain somatic symptoms, including headache, nausea, heartburn, breathlessness, palpitations and trouble walking [37]. In our study, among patients who complained of dizziness, the rate of functional limitation due to emotional problems and lack of emotional well being was higher than the rate among patients without dizziness. In addition, we found that 66% of the patients who complained of fatigue suffered from functional limitations due to emotional problems, while this percentage was much lower among those without fatigue.

A larger number of somatic complaints is associated with a greater possibility that the patient has an emotional problem [15,38]. According to Kroenke [6] the risk of an emotional disorder in a patient visiting a primary clinic increases with the number of somatic symptoms of which he or she complains. Our research supports this, too; there is a significant difference between lack of emotional well being and functional limitations due to emotional problems in patients who report two or more somatic symptoms and between those who report just one.

Most primary physicians were trained with a biomedical orientation, in which the main purpose of the visit is to rule out organic disease [21]. This medical model often leaves mental etiologies as a "diagnosis of exclusion" [2]. Therefore, patients who have mental illness or emotional distress, and complain of somatic symptoms, are usually diagnosed later than those who report psychological or social symptom [22,31,32,39]. Similarly, our research revealed that the medical records of only 25% of patients with functional limitations due to emotional problems or lack of emotional well being contained a mental/emotional diagnosis. At the same time, it is likely that not every injury to emotional well being translates into a diagnosable mental/emotional or psychiatric disease. Another point to take into account is that the physician may be reluctant to write such a diagnosis in the file based on the first visit.

The study has some limitations. Even with a "friendly" questionnaire, one of the weaknesses of our research is the low (41.8%) response rate, despite two telephone reminders. Nevertheless, this rate was close to that described in a broad study that examined the percentage of patients who responded to questionnaires and surveys in published research during the period 1985-1995 [40]. According to the study, only about 60% of patients respond to questionnaires mailed to them by their personal attending physician, and only about 50% respond to general surveys. Our questionnaire was sent to the patients from an unknown person, although with an accompanying letter from the HMO headquarters. There is also the possibility that some of the patients were not fluent in Hebrew or even illiterate as at least 20% of the HMO population is new immigrants to Israel. At the same time, the finding that the group of patients that returned the questionnaires is similar in all attributes to the group that did not return them does help to validate our data. A similar study was done in Scotland [41] using an electronic diary instead of questionnaires, but numbers were very small. Another limitation is the retrospective evaluation of symptoms. However, the work is strengthened by the use of a control group which is very similar to the research group. A further limitation was the lack of a review of the medical records of the patients in the control group for mental health diagnoses made during the follow-up year by the family physicians which would have allowed comparison with the research group.

## Conclusions

We conclude by stating that because of the frequency of non-specific somatic symptoms seen by the primary care physician, and the possibility of a "hidden" mental disease or at least emotional distress accompanying them, it is the physician's duty to make use of every available clue in order to diagnose these conditions as early as possible. Our study shows that headache, fatigue, dizziness and insomnia are especially related to lack of emotional well being and to functional limitations due to emotional problems. Despite this, additional research is needed to examine the relation of these somatic symptoms to a diagnosis of depression and anxiety.

## Declaration of competing interests

The authors declare that they have no competing interests.

## Authors' contributions

AM and EK conceived of the study. AM, AK and EK designed the study together. Data was collected by AK and EK and the analysis of the data was carried out by all five authors. Statistical analysis was performed by AK and JY. GB wrote the paper with help from AM, AK, JY and EK. All authors read and approved the final manuscript.

## Pre-publication history

The pre-publication history for this paper can be accessed here:

http://www.biomedcentral.com/1471-2296/12/77/prepub
